# Dynamics of Distraction: Competition among Auditory Streams Modulates Gain and Disrupts Inter-Trial Phase Coherence in the Human Electroencephalogram

**DOI:** 10.1371/journal.pone.0053953

**Published:** 2013-01-10

**Authors:** Karla D. Ponjavic-Conte, Dillon A. Hambrook, Sebastian Pavlovic, Matthew S. Tata

**Affiliations:** Department of Neuroscience, The University of Lethbridge, Lethbridge, Alberta, Canada; McGill University, Canada

## Abstract

Auditory distraction is a failure to maintain focus on a stream of sounds. We investigated the neural correlates of distraction in a selective-listening pitch-discrimination task with high (competing speech) or low (white noise) distraction. High-distraction impaired performance and reduced the N1 peak of the auditory Event-Related Potential evoked by probe tones. In a series of simulations, we explored two theories to account for this effect: disruption of sensory gain or a disruption of inter-trial phase consistency. When compared to these simulations, our data were consistent with both effects of distraction. Distraction reduced the gain of the auditory evoked potential and disrupted the inter-trial phase consistency with which the brain responds to stimulus events. Tones at a non-target, unattended frequency were more susceptible to the effects of distraction than tones within an attended frequency band.

## Introduction

In complex acoustic environments, listening selectively to one out of many sources of input can present a significant challenge to the human auditory system. In the auditory modality these sources of input are often referred to as streams, and parsing the environment for such streams has been referred to as auditory scene analysis [1]. Competing streams can disrupt perception of a target stream, even when those streams occupy distinct channels at the sensory periphery. This phenomenon has been conceptualized as a failure of attentional selectivity [2], [3], but also in the context of auditory masking [4], [5]. Here we adopt the use of the broad but intuitive term *distraction* [6] to describe perceptual competition among auditory streams. Several decades of psychophysical research have described the perceptual consequences of distraction, yet little is known about the how the neuro-electric representation of task-relevant stimuli changes when a distractor is present in the auditory scene. The present study reveals that distracting speech attenuates the gain and disrupts the temporal fidelity of cortical responses to sounds in the auditory scene.

Probably the best example of real-world distraction is the “two-talker” problem. In the two-talker problem, speech perception is impaired when another stream of speech is mixed into the signal. The extreme case is the canonical “cocktail party” in which many independent streams are mixed. The “two-talker” problem differs markedly from paradigms commonly used to study auditory distraction in the laboratory. Such paradigms study the physiological correlates of unusual discrete events happening in the auditory scene [7] but the objective of our study was to investigate the physiological correlates of distraction when there is a continuously competing stimulus in the auditory scene.

The decrement in perception observed in the two-talker problem has been called auditory informational masking [8]. Information masking occurs when a target signal is embedded in a competing signal that impairs target detection, discrimination or intelligibility of speech even when the target and masker do not overlap in frequency [9]. The parameters that affect informational masking are similar to those that affect the allocation of attention in any complex display. For example, informational masking is particularly strong in dynamic displays, and when the target sound and masker sounds are similar to each other (that is, when the target fails to “pop out”) [9], [10]. In this sense, auditory informational masking is loosely analogous to object substitution masking in *vision*, which occurs when a visual target is embedded in a complex display [11], [12], [13]. The relationship between informational masking, distraction and selective attention remains poorly characterized in the literature although the term “distraction” has been used to define “informational masking” by Durlach and colleagues [6]. Thus informational masking in the two-talker situation is a good context in which to study attention and distraction.

The presence of task-irrelevant speech or music in the auditory scene is well-known to attenuate and delay the N1 component of the auditory Event-Related Potential, or its magnetic counterpart the N1m, when it is evoked by transient probe stimuli [14], [15], [16], [17], [18]. For example, Hari & Makela [16] showed that music, speech, and to a lesser degree intermittent noise, presented to the ipsilateral ear, delayed and attenuated the N1m response to 25 ms broadband pulses. The reason for this effect in the presence of a competing auditory stream is unknown, however the phenomenon is well-aligned with studies of selective attention: The N1 component evoked by attended stimuli is typically larger relative to ignored stimuli [19], [20]. This effect only develops after listeners have maintained selection of the target stream for a period of many seconds [21], [22]. It does not occur when attention is reoriented on a moment-by-moment basis as would be expected when a competing stream is present [23], [24], [25] but see [26] for evidence to the contrary. Thus, there is a consistent picture of attenuation of early ERP components in both informational masking and attention orienting paradigms, but the mechanism underlying such attenuation remains unknown.

Ponjavic-Conte et al. (2012) replicated the attenuation of the N1 ERP due to distraction [27]. They proposed two theories to account for this effect. One theory is that distraction transiently captures attention away from the target stream, thereby reducing the boost in sensory gain afforded by sustained attention. This account follows from the “sensory gain-control” theory, which holds that attention modulates the gain of fixed-latency responses in sensory systems [28]. That is, cells that encode to-be-attended stimuli show a larger response than cells that encode features of unattended stimuli [29], [30]. Thus, by breaking sustained attention, a distracting stream could attenuate and delay early ERP components evoked by target stimuli. Importantly, in this theory of distraction, the fixed-latency ERP remains time-locked to the evoking stimuli, but it is attenuated in amplitude and delayed by a constant latency in time. We refer to this theory below as the Attenuate-and-Delay model.

In contrast, Ponjavic-Conte et al. [27] suggested that distraction might disrupt the temporal fidelity of evoked responses, such that their phase consistency over successive trials is reduced. Here we suggest the term *Distraction Decoherence* and describe it as a phenomenon of signal jitter. Ponjavic-Conte et al. [27] based their idea on the observation that inter-trial phase coherence in the theta EEG band was reduced when a speech masker was present in the scene, relative to when a broadband noise masker was present. Inter-trial phase coherence is a measure of the temporal similarity of brain electrical signals over successive trials. Thus the measure can, in principle, reveal differences in the degree of phase consistency across different stimulus configurations and cognitive tasks.

Other work is broadly consistent with the theory of *Distraction Decoherence*. For example, Tiitinen et al. [31] suggested that selective attention could sharpen the temporal fidelity of the 40 Hz steady-state response. Low & Strauss [32] showed that responses to auditory targets exhibit more inter-trial phase consistency than responses to non-targets. Substantial literature has recently emphasized the effect of selective attention on oscillatory signals in the EEG [33], [34], [35], [36], [37]; in addition, the phase dynamics of cortical oscillations is thought to be a critical factor in the computational architecture of the cortex [38]. The possible disruption of the inter-trial phase consistency of early auditory responses due to distraction is therefore of particular theoretical importance.

Ponjavic-Conte et al. [27] found that continuous speech in the auditory scene attenuated the N1 and reduced inter-trial phase coherence in the theta band. In the current study we sought to replicate these results in a pitch-discrimination task. However, since the inter-trial phase coherence measure is sensitive to changes in the signal-to-noise ratio, a reduction in sensory gain might also appear as a reduction in inter-trial phase coherence. Thus, we simulated both the Attenuate-and-Delay model and the Distraction Decoherence model. Our empirical data match aspects of both simulations suggesting that the early ERP is both attenuated in gain and jittered in time when a competing speech distractor is present. The result of this simulation is of interest more broadly because it shows that, in principle, any apparent attenuation of an evoked signal averaged over successive trials can be explained by phase decoherence rather than gain modulation.

## Experiment One

Ponjavic-Conte et al. (2012) used a temporal discrimination task in which participants discriminated the duration of a brief silent gap in a burst of noise [27]. We considered that the temporal effects of distraction evident in the EEG might be unique to this duration-discrimination task so in the present study we instead used a pitch-discrimination task. We also included an “off-band” unattended non-target tone to investigate the role of top-down attentional set. Our first goal was to establish whether speech distraction has a measureable effect on task performance in a pitch-discrimination task.

### Methods

#### Ethics Statement

All participants provided informed written consent. Procedures were in accordance with the Declaration of Helsinki and were approved by the University of Lethbridge Human Subjects Review Committee.

Fifteen undergraduates from the University of Lethbridge were recruited and participated for course credit. Participants were neurologically normal and reported normal hearing. Participants were also screened with the World Health Organization Adult Attention-Deficit Hyperactivity Disorder (ADHD) self-report scale (ASRS) [39]. Three participants were excluded from the analysis for not following task instructions (their false alarm rate was 3 standard deviations outside the mean in both low- and high-distraction). Thus, 12 participants contributed to the data analysis (9 females; one left-handed; average age: 21.3).

Stimuli were presented on an Apple Mac Mini with sound attenuating headphones (approx. 30 dB attenuation); volume was individually adjusted to a comfortable volume. Auditory stimuli were created using MATLAB (MATLAB version 7.10.0; The Mathworks Inc., 2010, Natick, Massachusetts, USA) and controlled by a program custom coded using Apple Computer’s Core Audio framework (Mac OS 10.6). Sounds were panned equally to both left and right ears such that they were localized to the midline.

Each session consisted of 26 blocks of 1.2 minute duration in which two different streams of sound (a target stream and a distraction stream) were presented simultaneously to both ears. The target stream consisted of two target tones (target-high: 1000 Hz; target-low: 975 Hz) that were to be attended and one non-target tone (600 Hz) that was to be unattended; all tones were 200 ms in duration. In each block, nine target-high, nine target-low and 18 non-target tones were presented in a randomized order with an inter-stimulus interval of 1.94 seconds +/−250 ms of jitter. The distraction stream consisted of one of two types of stimuli. The low-distraction condition was continuous broad-band noise. The high-distraction condition was randomly selected segments of audio books consisting only of the voice of a single reader (i.e. no sound effects). The root mean square amplitude of each low-distraction stimulus was matched to that of a high-distraction stimulus. In each session, 13 low-distraction and 13 high-distraction blocks were presented pseudorandomly.

Participants were instructed to attend to the target-high and target-low tones so that they could discriminate between them, while ignoring the much lower non-target tone along with the distracting noise or speech. The required response was to press the up arrow key for the target-high tone and press the down arrow key for the target-low tone, and to withhold response for the non-target tone. Maximum response time allotted per trial was 750 ms. A response was considered an accurate hit if the participant discriminated correctly between the target-low and target-high tones. Thus, discrimination accuracy was measured as a percentage of correct target-present trials. Possible behavioural data outcomes are depicted in Table 1. The effect of distraction (high vs. low) on mean response times, discrimination accuracy, false alarms, correct-rejections and misses were assessed by two-tailed t-tests.

**Table 1:** Behavioural data outcomes.

	Participant’s Response		
Auditory Stimulus	Up Arrow	Down Arrow	None
High-Pitch Target	Accurate Hit	Inaccurate Hit	Miss
Low-Pitch Target	Inaccurate Hit	Accurate Hit	Miss
Non-Target	False Alarm	False Alarm	Correct Rejection

Possible behavioural data outcomes are depicted. Discrimination accuracy between the two tones within the target frequency band (975 Hz and 1000 Hz) was calculated as the number of correct responses divided by the total number of hits.

### Results

The high-distraction condition decreased listener ability to discriminate accurately between the target-low and target-high tones (Mean low-distraction: 0.771, SD = 0.202; Mean high-distraction: 0.728, SD = 0.199; t_11_ = 2.426; P = 0.034). Participants tended to make more “false alarm” responses to the low-pitch non-target tone (Mean low-distraction: 0.010, SD = 0.010; Mean high-distraction: 0.030, SD = 0.027) and were more likely to miss the high-pitched target tones in the high-distraction condition (Mean low-distraction: 0.250, SD = 0.157; Mean high-distraction 0.270, SD = 0.173); but these effects were not significant. There was also no effect of distraction condition on response times (Mean low-distraction: 548.6, SD = 44.6; Mean high-distraction: 552.3, SD = 48.6).

### Discussion

Experiment One confirmed that the experimental paradigm of distraction used by Ponjavic-Conte et al. (2012) extends also to pitch discrimination and is consistent with a large body of literature in the domain of informational masking. The presence of task-irrelevant speech in the auditory scene impaired performance of a difficult pitch discrimination. Experiment Two considers the neurophysiological correlates of distraction.

## Experiment Two

Distraction in Experiment One impaired pitch discrimination. Experiment Two considers the neurophysiological basis for this effect of distraction.

### Methods

Task parameters were as in Experiment One except that sounds were presented in free field by a Mac Pro with a firewire audio interface (M-Audio Firewire 410). Participants sat in front of two near-field studio monitors (Mackie HR624 MK-2) arranged vertically (one monitor played the target stream; the other played the distraction stream). Participants were seated in a dimly lit and sound attenuated room.

Nineteen undergraduates participated in the study for course credit. Two were excluded due to excessive artifact in the EEG (deflections of greater than +/−120 µV) and two because they screened positive for ADHD on the ASRS; thus 15 participants were included in the analysis (11 female; all right-handed; average age: 22.5). Procedures were in accordance with the Declaration of Helsinki and were approved by the University of Lethbridge Human Subjects Review Committee; all participants gave written informed consent.

The EEG was recorded with 128 Ag/Ag-Cl electrodes in an elastic net (Electrical Geodesics Inc., Eugene, OR, USA). Scalp voltages were recorded with a 500 Hz sampling rate and impedances were maintained under 100 kilo-ohms. Data were analyzed using the BESA software package (Megis Software 5.3, Grafelfing, Germany). The EEG was first visually inspected for bad electrodes and a small number of electrodes (10 or less) per participant were replaced with an interpolated signal.

ERP waveforms were time locked to target and non-target tones [high-pass (0.5 Hz, 12 dB/octave); low-pass (30 Hz, 24 dB/octave) zero-phase Butterworth filters; re-referenced to a standard 10–10 average-reference montage with a 200 ms pre-stimulus baseline]. Epochs containing artifact (deflections of greater than +/−120 µV) were rejected. Participants had few miss and false alarm trials, thus after artifact rejection only accurate responses to targets (i.e. hits) and correct-rejection of non-targets (i.e. correct-rejections) had enough epochs (>25) to be analyzed across all participants. We refer to these conditions below as “Attended Hits” and “Unattended Correct-rejections”. The average number of trials per participant per condition after artifact rejection were as follows: Attended Hits under low-distraction: 118; Attended Hits under high-distraction: 117; Unattended Correct-rejections under low-distraction: 165; Unattended Correct-rejections under high-distraction: 169.

The N1 peak was identified at electrode Cz for all conditions at latencies ranging from 118–122 ms (Attended Hits low-distraction: 118 ms; Attended Hits high-distraction: 120 ms; Unattended Correct-rejections low-distraction: 120 ms; Unattended Correct-rejections high-distraction: 122 ms). For statistical comparisons, the mean amplitude of the N1 peak for all conditions was computed within a window spanning 6 ms on either side of 120 ms (without filtering) and by using an average reference. A repeated-measures ANOVA with two levels of the factor Distraction (low/high distraction) and two levels of the factor Frequency Selection (target/non-target) was performed on N1 mean amplitudes. Difference waves were computed for differences due to distraction and viewed in an isopotential map by subtracting the ERP waveforms in the high-distraction condition from waveforms in the low-distraction condition.

In order to assess the possibility that differences in evoked responses during low- and high-distraction could be due to increased energetic masking by the speech distractor relative to the broad-band noise distractor, high-distraction trials were reclassified as being high-energy or low-energy based on the spectrogram of the speech distractor at the moment of target/non-target presentation. The power spectral density of the speech distractor was calculated using a short Fourier transform for the duration of each target tone (200 ms), centered at the tone frequency. If the power spectral density of the speech distractor for a particular trial was greater than the grand mean power spectral density for the broad-band noise distractor at that frequency, then that trial was reclassified as being high-energy/high-distraction; if the power spectral density for a trial was less than the grand mean power spectral density of the broad-band noise distractor, the trial was reclassified as being low-energy/high-distraction. Reclassifying trials in this way allowed the effect of distraction to be dissociated from energetic masking by the speech distractors. The proportion of reclassified high-energy/high-distraction trials to low-energy/high-distraction trials for Unattended Correct-rejections was 28.2 to 71.8 and the proportion of reclassified high-energy/high-distraction trials to low-energy/high-distraction trials for Attended Hits was 16.3 to 83.7. In the case of the target tones (975 or 1000 Hz) there were very few trials in which the speech masker exceeded the energy of the noise masker at the moment of the target; so few that we found we could not even generate a meaningful event-related potential. Therefore only grand-averaged ERP waveforms for Unattended Correct-rejections in high-energy/high-distraction, low-energy/high-distraction and low-distraction were created for visualization [high-pass (0.5 Hz, 12 dB/octave); low-pass (30 Hz, 24 dB/octave) zero-phase Butterworth filters; re-referenced to a standard 10–10 average-reference montage with a 200 ms pre-stimulus baseline]. For statistical comparisons, two-tailed t-tests were performed on N1 mean amplitudes (within a window spanning 6 ms on either side of 120 ms (without filtering) and by using an average reference).

The raw EEG was transformed into time-frequency space using complex demodulation as implemented in BESA 5.3 [40] between 4 and 46 Hz, from −200 to 800 ms, and exported in 2 Hz/25 ms sample bins. The time-spectral data for each participant for Attended Hits and Unattended Correct-rejections in both low- and high-distraction conditions was then exported from BESA and imported into Matlab. Grand-averaged Inter-trial phase coherence, Total Power, Induced Power and Evoked Power at electrode Cz were calculated for Attended Hits and Unattended Correct-rejections in low- and high-distraction conditions.

Inter-trial phase coherence (ITC) was calculated by the following
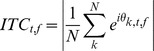
where N is equal to the number of trials, and θ is the phase of trial k at a given frequency (f) and time (t). Inter-trial phase coherence is a measure of the similarity of the phases of signals over many repetitions. The values of inter-trial phase coherence range from 0 to 1 with 1 meaning perfect phase consistency across trials.

Total power, induced power and evoked power were calculated by the following. First the total power in the pre-stimulus (−200 ms to −100 ms) baseline was computed:
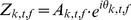


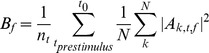
Where n_t_ is the number of time bins before t = −100 ms, A_k,t,f_ is the coefficient of the complex valued result (Z_k,t,f_) of the complex demodulation for trial k, frequency f, and time t; B_f_ is the baseline power for a given frequency f. Power was then computed relative to the baseline:



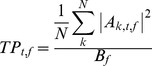


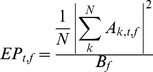


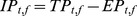
Where TP_t,f_ is the total power percent change from baseline for a given time t, and frequency f; EP_t,f_ is the percent change in power that is evoked (i.e. phase-locked) and IP_t,f_ is the non-phase locked change in power from the baseline. Both evoked and induced power represent changes in power that are time locked to the onset of a stimulus but evoked power and induced power differ in their phase relationship to the stimulus. Evoked power is phase locked to stimulus onset, thereby capturing phase-consistent power across trials. By contrast, induced power does not capture phase-locked power. Instead, it is a measure of the power of oscillatory activity with no phase consistency across trials. Both evoked power and induced power were calculated to determine what proportion of the total change in power in single trials was phase-locked to the stimulus. Since by definition evoked power and induced power sum to equal total power, given a constant total power, evoked power and induced power must vary inversely.

We compared the difference between low- and high-distraction for inter-trial phase coherence for Attended Hits and Unattended Correct-rejections with a random-sample permutation method and applied a False-Discovery Rate (FDR) correction method to control for multiple comparisons across time and frequency bins [41]. A surrogate distribution was built for each participant by randomly shuffling trials between low- and high-distraction conditions (thus preserving the original number of trials in each condition) and then by re-computing the difference between conditions. This process was repeated 40 000 times for each participant to create a surrogate distribution of differences. The surrogate distributions were then averaged to produce a grand-average surrogate distribution of differences. The original grand-average difference was then compared to this surrogate distribution of differences, and a two-tailed P-value (2 x the proportion of surrogate differences that fell beyond the observed difference) for each time/frequency bin was obtained. Differences between low- and high-distraction conditions in total, evoked and induced power were compared using the same procedure.

In order to further investigate the inter-trial phase coherence difference at the N1 latency between low- and high-distraction for Attended Hits and Unattended Correct-rejections, we chose to focus our analysis on the 150 ms/6 Hz time-frequency bin. This time-frequency bin was chosen because it captured most of the inter-trial phase coherence difference between low- and high-distraction for both Attended Hits and Unattended Correct-rejections. Since the raw EEG was transformed into time-frequency space in 25 ms/2 Hz samples, the 150 ms/6 Hz time-frequency bin also captures activity occurring around the observed N1 latency (118–122 ms). Radial histogram plots of phase angle (in degrees) and the proportion of trials that fell within each phase angle bin were constructed for the 150 ms/6 Hz time-frequency bin. These were computed separately for each subject and then averaged across subjects. In order to examine the distribution of mean phases for low- and high-distraction at the 150 ms/6 Hz time-frequency bin, a Watson-Williams test was performed to compare the mean phase angles of low- vs. high-distraction trials. This was followed by a Kruskal-Wallis one-way analysis of variance that tested the concentration factor of phase between low- and high-distraction conditions [42].

### Results

As in Experiment One, high-distraction significantly reduced listener accuracy in discriminating between the two tones within the target frequency band (975 Hz and 1000 Hz) (Mean low-distraction: 0.784, SD = 0.194; Mean high-distraction: 0.732, SD = 0.170) as was assessed by a two-tailed t-test (t_14_ = 2.421; P = 0.030). There was a non-significant trend for participants to make more misses during high-distraction (Mean low-distraction: 0.296, SD = 0.118; Mean high-distraction: 0.315, SD = 0.106). There was no effect of distraction on response times (Mean low-distraction: 579.4, SD = 48.0; Mean high-distraction: 575.3, SD = 46.1).

**Figure 1:**
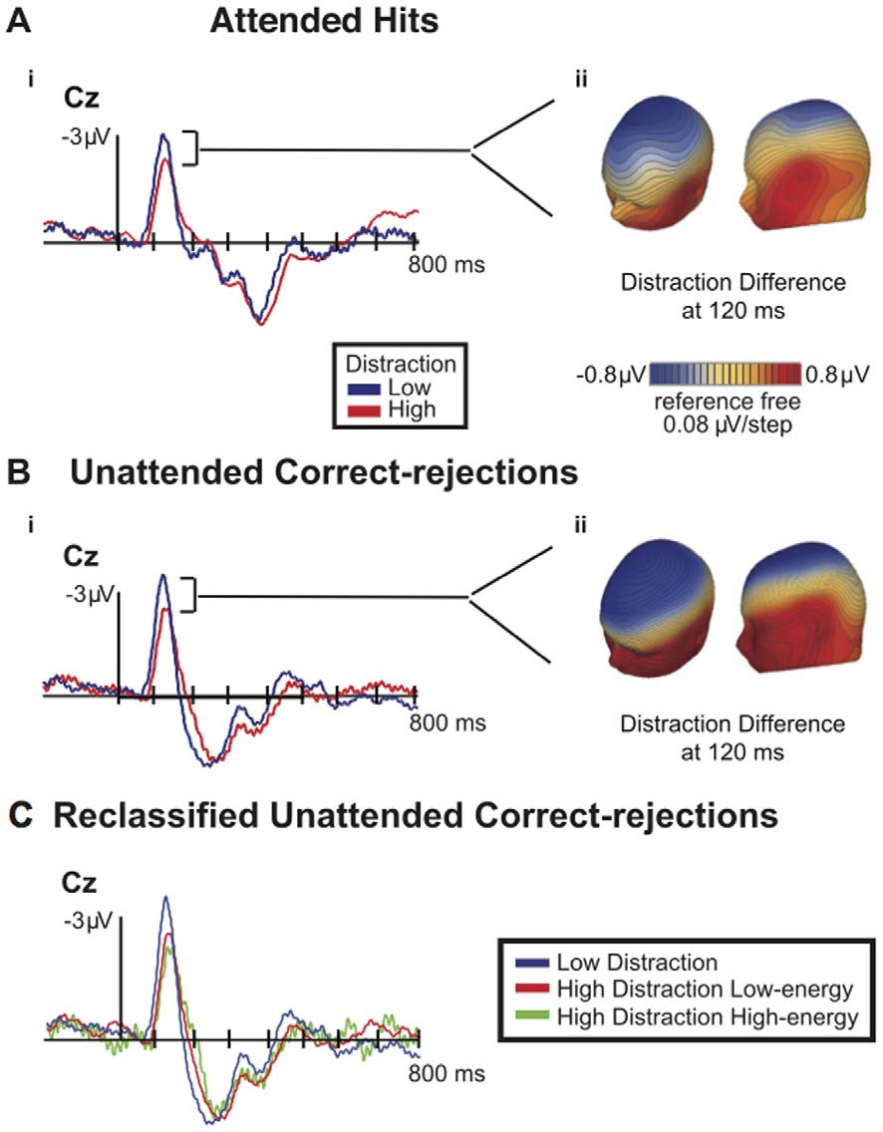
ERP waveforms evoked by target-present hits (Attended Hits) and by target-absent correct-rejections (Unattended Correct-rejections). **1A)** (**i**) ERP waveforms evoked by Attended Hits in low- and high-distraction conditions. The N1 was maximal at Cz in low-distraction at 118 ms and in high-distraction at 120 ms. It was attenuated in high-distraction (t_14_ = 2.649; P = 0.019). **1A** (**ii**) Isopotential maps of Attended Hits N1 peak difference between low- and high-distraction at 120 ms. **1B)** (**i**) ERP waveforms evoked by target-absent correct rejections (Unattended Correct-rejections) in low- and high-distraction conditions. The N1 was maximal at Cz in low-distraction at 120 ms and in high-distraction at 122 ms. It was attenuated in high-distraction (t_14_ = 2.387; P = 0.032). **1B** (**ii**) Isopotential map of Unattended Correct-rejections N1 peak difference between low- and high-distraction at 120 ms. **1C** Reclassified ERP waveforms evoked by target absent correct-rejections (i.e. Unattended Correct-rejections) in high-energy/high-distraction, low-energy/high-distraction and low-distraction at electrode Cz. No difference was found between high-energy and low-energy high-distraction trials (t_14_ = 0.022; P = 0.983). Comparisons between low-energy/high-distraction and low-distraction revealed a significant difference (t_14_ = 2.336; P = 0.035). Thus, N1 attenuation in high-distraction is not due to energetic masking associated with the speech distractor.

We observed a prominent N1 peak in the low-distraction condition and attenuation of this peak in the high-distraction condition for both Attended Hits (Mean low-distraction: −2.810, SD = 1.235; Mean high-distraction: −1.982, SD = 1.251) and Unattended Correct-rejections (Mean low-distraction: −3.253, SD = 1.293; Mean high-distraction: −2.285, SD = 1.331) (Fig. 1A(i) and Fig. 1B(i)). A two-way repeated measures ANOVA on N1 mean amplitudes revealed a main effect of frequency selection (i.e. Attended Hits vs. Unattended Correct-rejections) (F_(1,14)_ = 5.730; P = 0.031; ε = 1.000) as well as a main effect of distraction (i.e. high vs. low) (F_(1,14)_ = 8.404; P = 0.012; ε = 1.000), but no interaction (F_(1,14)_ = 0.142; P = 0.712; ε = 1.000). The isopotential maps revealed a fronto-central focus of the N1 difference (Fig. 1A(ii) and 1b(ii)) with a polarity reversal at temporal sites consistent with generator(s) on the supratemporal plane. This was apparent for both Attended Hits and Unattended Correct-rejections.

Grand-averaged ERP waveforms for Unattended Correct-rejections in high-energy/high-distraction, low-energy/high-distraction and low-distraction and be viewed in Figure 1C. There was no difference of N1 mean amplitudes between high-energy/high-distraction and low-energy/high-distraction trials (t_14_ = 0.022; P = 0.983). However, the distraction effect is still evident when low- and high-distraction trials are equated for energy (i.e. low-energy/high-distraction and low-distraction trials) (t_14_ = 2.336; P = 0.035).

**Figure 2:**
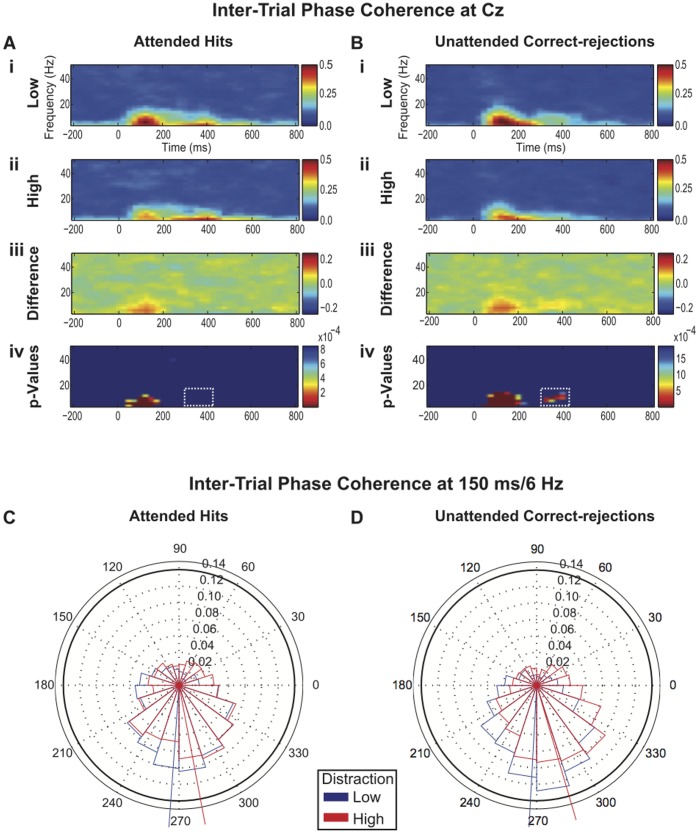
Inter-trial phase coherence and Phase Distributions. **2A)** Time-frequency plots of grand-averaged Inter-trial phase coherence at electrode Cz for Attended Hits in low (**i**) and high (**ii**) distraction. (**iii**) Time-frequency plot and (iv) FDR thresholded map of the differences between distraction conditions (low minus high) in Inter-trial phase coherence **2B)** Time-frequency plots of grand-averaged inter-trial phase coherence at electrode Cz for Unattended Correct-Rejections in low (**i**) and high (**ii**) distraction. (**iii**) Time-frequency plot and (**iv**) FDR thresholded map of the differences between distraction conditions (low minus high) in inter-trial phase coherence. There was a decrease of theta/alpha inter-trial phase coherence around the N1 latency in high-distraction for both Attended Hits and Unattended Correct-rejections. There was a decrease of theta and alpha inter-trial phase coherence for Unattended Correct-rejections (but not Attended Hits) at approximately 300 to 400 ms post-stimulus in high-distraction. **2C)** Grand-averaged radial histograms of phase angle distributions in the 150 ms/6 Hz time-frequency bin in low- and high-distraction for Attended Hits. Mean phase angles for low- and high-distraction are indicated by the blue and red lines, respectively. The distribution of phase angles was rotated (delayed) by distraction. The difference in mean phase angles was marginally significant (F_(1,14)_ = 3.06; P = 0.09) and the difference in phase concentration was significant (c^2^ (1, n = 15) = 4.56; P = 0.03). **2D)** Grand-averaged radial histograms of phase angle distributions for the 150 ms/6 Hz time-frequency bin in low- and high-distraction for Unattended Correct-rejections. The difference in mean phase angles and phase concentrations were both significant (F_(1,14)_ = 12.35; P = 0.0015) and (c^2^ (1, n = 15) = 5.11; P = 0.02), respectively, for Unattended Correct-rejections. Note that high-distraction in both Attended Hits and Unattended Correct-rejections appears to both broaden and shift the distribution of phases of 6 Hz theta band signals.

As predicted, distraction (high vs. low) significantly reduced theta/alpha band inter-trial phase coherence around the N1 latency for Attended Hits; this effect was also evident for Unattended Correct-rejections (Fig. 2A; Fig. 2B). In addition to reduced inter-trial phase coherence around the N1 latency, we also observed a later reduction in inter-trial phase coherence approximately 300 to 400 ms post-stimulus in the theta/alpha EEG band (4 to 12 Hz) but only for Unattended Correct-rejections (Fig. 2A(iv); Fig. 2B(iv)). This later reduction of inter-trial phase coherence for Unattended Correct-rejections also passed FDR correction for multiple paired comparisons (Fig. 2B(iv)). Eight time-frequency bins (between 300 to 400 ms and 8 to 12 Hz) passed FDR correction for inter-trial phase coherence of Unattended Correct-rejections with p-values ranging from 0.00005 to 0.00085, whereas no time-frequency bins passed FDR correction of inter-trial phase coherence for Attended Hits (p-values ranged from 0.4076 to 0.8944). Discussion of total, evoked and induced power is taken up in Experiment Three.

As evidenced by the radial histogram phase plots of the 150 ms/6 Hz time-frequency bin (Fig. 2C; Fig. 2D), Attended Hits and Unattended Correct-rejections exhibited different phase distributions at this frequency and latency depending on the level of distraction. The Watson-Williams test for different mean phase angles across distraction conditions found that the theta (6 Hz) phase distribution on high-distraction trials was significantly lagged (rotated counter-clockwise) (F_1,14_ = 12.35; P = 0.0015) for Unattended Correct-rejections at the 150 ms latency. Attended Hits also showed the same trend (F_1,14_ = 3.06; P = 0.09). Kruskal-Wallis one-way analysis of variance on the concentration of phase at the 150 ms/6 Hz time-frequency bin for low- and high-distraction conditions found a significant effect of distraction for both Attended Hits (c^2^ (1, n = 15) = 4.56; P = 0.03) and Unattended Correct-rejections (c^2^ (1, n = 15) = 5.11; P = 0.02). The effect of distraction (high vs. low) on mean concentration factor was larger for the Unattended Correct-rejection condition (Attended Hits low-distraction: 1.1005; Attended Hits high-distraction: 0.7732; Unattended Correct-rejections low-distraction: 1.345; Unattended Correct-rejections high-distraction: 0.902).

### Discussion

Previous investigations of competition among auditory streams have revealed that ERP components such as the N1 peak are attenuated and delayed by task-irrelevant distraction [14], [16], [17], [18], [27]. The modulation of the N1 component apparent in Figure 1 is consistent with this work. Furthermore, the reduction in inter-trial phase coherence evident in Figure 2, replicates the results reported by Ponjavic-Conte et al. [27]. The counterclockwise rotation of phase at the 6 Hz theta band during high-distraction (Fig. 2C; Fig. 2D) is also reflected in the latency shift of the N1 peak (Fig. 1A(i); Fig. 1B(i)). Reduced inter-trial phase coherence and broadening of the phase distribution evident in the phase histograms suggests that temporal jitter across trials might account for the attenuation of the N1 component.

When designing the stimuli and task for the present study, we adjusted the root mean square amplitude of each noise distractor to match one of the speech distractors. This resulted in the speech and noise stimuli being approximately matched in apparent loudness. However, speech and broadband noise have very different spectrotemporal properties. Speech is characterized by a high degree of spectrotemporal dynamics such as sharp discontinuities in energy and pitch, whereas broadband noise is relatively constant. The target and non-target tones were 200 ms in duration. Thus for some presentations of these stimuli, the speech distractor might have contained relatively high energy at the same frequencies. In such cases, the distracting effect of speech was confounded with energetic masking. Energetic masking occurs when a continuous tone or noise acts as a masker because of its spectral overlap with the target; it is distinct from informational masking in which masking occurs when a target signal is embedded in a competing signal that impairs target detection, discrimination or intelligibility of speech even when the target and masker do not overlap in frequency [6], [9].

To address this confound, a second analysis on N1 mean amplitudes was done to assess whether the N1 attenuation during high-distraction was due to increased energetic masking by the speech distractor. High-distraction trials were reclassified as being high-energy/high-distraction or low-energy/high-distraction. The N1 mean amplitude analysis revealed that even when equated for energy, distraction (high vs. low) still attenuated the N1 (Fig. 1C). Thus N1 attenuation observed in high-distraction can be dissociated from the energetic masking confound and instead the present results can likely be considered in the context of auditory informational masking.

A secondary goal was to test whether attention on a target stream would protect brain responses from the effects of distraction. We found that high-distraction reduces inter-trial phase coherence at the theta and alpha EEG bands at latencies beyond the N1 (300 to 400 ms), but only for Unattended Correct-rejections. Responses to attended targets appear to be protected from this later distraction effect, but it is possible that our test simply lacked the statistical power to find these effects in the Attended Hit condition. Our data therefore suggest that one effect of top-down attentional selection is to protect the phase stability of theta/alpha responses under high-distraction. We speculate that maintenance of good temporal-fidelity might be critical for early sensory systems to contribute information to response-planning and memory processes in other brain regions [38]. Alternatively, it is possible that the presence of a phase-locked P300 component in the ERP for Attended Hits but not Unattended Correct-rejections might have masked a difference in inter-trial phase coherence and evoked power at the 300 to 400 ms post-stimulus latency range.

Although the inter-trial phase coherence measure is sensitive to the phase consistency of signals across trials, it is also sensitive to the amplitude ratio of signal to noise. Thus, reducing the amplitude of a signal typically also reduces its inter-trial phase coherence value unless the noise floor is also reduced. Therefore, the inter-trial phase coherence modulation observed in high vs. low-distraction in Experiment Two and in Ponjavic-Conte et al. [27] does not unequivocally indicate a reduction of phase consistency. An Attenuate-and-Delay model in which fixed-latency peaks are simply reduced in amplitude could also account for these data. A recent study by David et al. [43] examined this ambiguity in interpreting the inter-trial phase coherence value. Their simulations suggested that signal jitter has the effect of shifting power from the phase-locked “evoked” signal onto the non-phase-locked “induced” signal. Evoked power is the portion of signal that is time-locked to the event of interest. It is the basis of the event-related potential (i.e. all the power revealed by the ERP waveform is evoked). By contrast, induced power is the portion of power in a signal that is not phase-locked. By definition (see methods above), induced power can be computed as the difference between total power and evoked power [43]. Experiment Three was designed to explore this interaction and seek a test that can reliably distinguish between an Attenuate-and-Delay model and a Distraction Decoherence model of distraction.

## Experiment 3

Distraction in Experiment Two was associated with an attenuation of the N1 component and a reduction in inter-trial phase coherence. In Experiment Three we simulated both the Attenuate-and-Delay model and the Distraction Decoherence model to consider which model best fits our empirical data.

Here we explicitly describe two models to account for the reduction and latency shift observed in the N1 during distraction. The Attenuate-and-Delay model is based on the notion that attention boosts the gain of the auditory cortex response relative to unattended stimuli [28], and that distraction aborts this amplification. To account for the latency shift in our results (and others) this model must also include a delay of a fixed latency in the ERP signal. By contrast, the Distraction Decoherence model proposes that temporal variability in the evoked signal accounts for both the attenuation and latency shift evident in the N1 peak. In this model, time locking between the evoked signal and the sensory event that triggers it becomes less precise under conditions of distraction. The evoked signal under low-distraction thus represents a “best case” latency to which a random phase lag is added. Importantly, in the Distraction Decoherence model the amplitude of the ERP signal on individual trials remains constant, but the latency is randomly distributed.

### Methods

**Figure 3:**
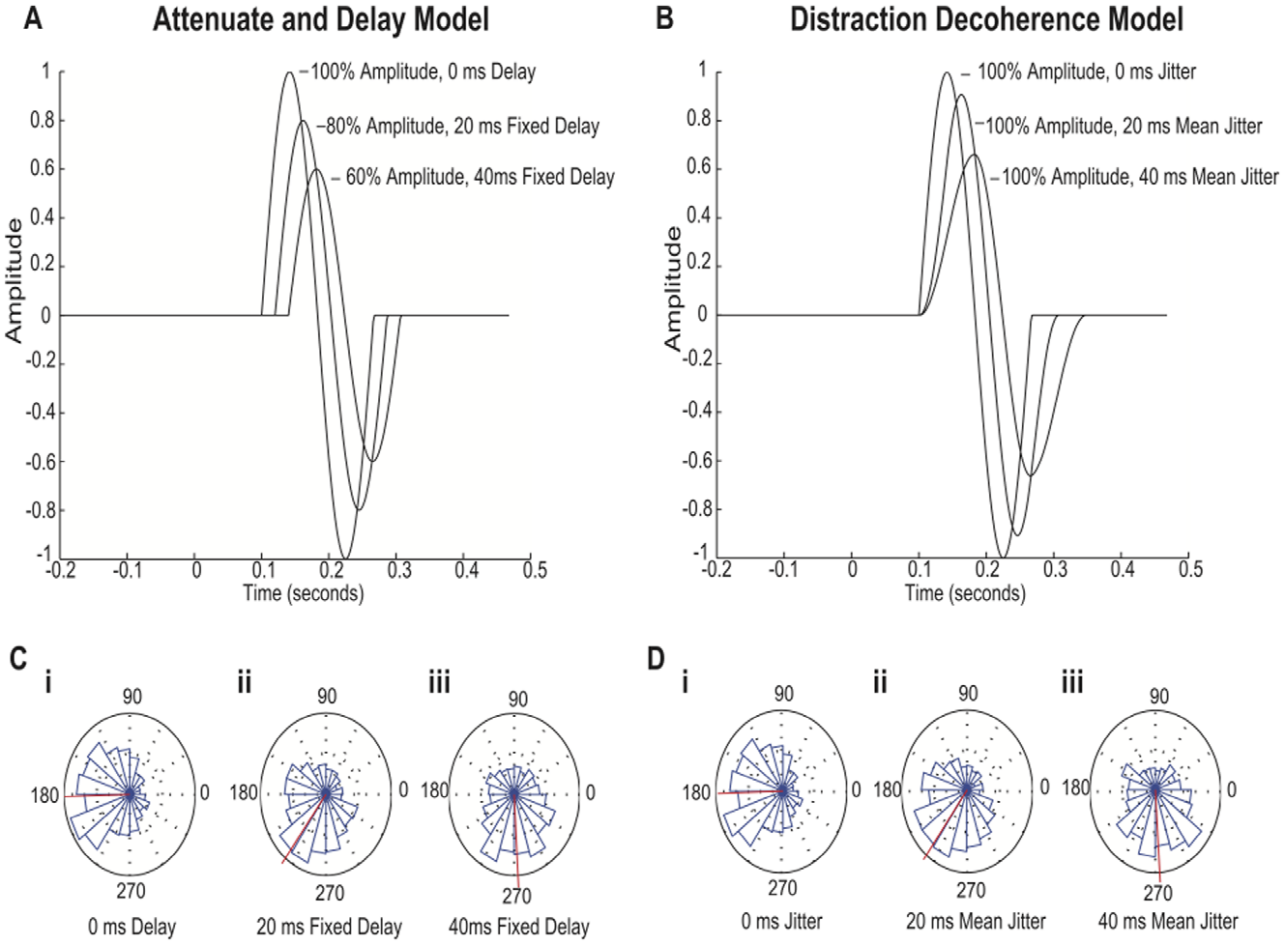
Simulated ERP Waveforms and Phase Distributions. **3A)** Attenuate and Delay model. A single-cycle 6 Hz (theta band) sinusoidal waveform embedded in 1/f noise (omitted for clarity) was simulated for three levels of modulation: 100% amplitude/0 ms fixed delay; 80% amplitude/20 ms fixed delay; 60% amplitude/40 ms fixed delay. In this model the waveform on individual trials within each condition varied in amplitude but had fixed latencies. **3B)** Distraction Decoherence Model. A single-cycle 6 Hz (theta band) sinusoidal waveform embedded in 1/f noise (omitted for clarity) was simulated for three levels of jitter: 100% amplitude/no jitter; 100% amplitude/20 ms mean jitter; 100% amplitude/40 ms mean jitter. In this model, the waveform on individual trials was always 100% amplitude for each condition but varied in latency. **3C)** Radial phase distributions and mean phase at the N1 latency for the Attenuate and Delay model (**i**) 100% amplitude/0 ms delay; (**ii**) 80% amplitude/20 ms fixed delay; (**iii**) 60% amplitude/40 ms fixed delay. Mean phase angles are indicated by the red lines. **3D)** Radial phase distributions and mean phase at the N1 latency for the Distraction Decoherence Model. (**i**) 100% amplitude/0 ms delay; (**ii**) 100% amplitude/20 ms mean jitter; (**iii**) 100% amplitude/40 ms mean jitter. Note that in both models, the distribution of phases is broadened and rotated counter-clockwise (i.e. delayed).

The ERP waveform was modeled as a single-cycle 6 Hz (theta band) sinusoidal waveform embedded in 1/f noise. The Attenuate-and-Delay model (Fig. 3A) was simulated for three levels of modulation: 100% amplitude/0 ms delay; 80% amplitude/20 ms fixed delay; 60% amplitude/40 ms fixed delay. Thus the ERP signal was attenuated and shifted in time by a fixed latency. The Distraction Decoherence model (Fig. 3B) was simulated for three levels of signal jitter. In one simulation the signal was un-jittered (i.e. the signal was identical to the 100% amplitude/0 ms delay condition in the Attenuate-and-Delay model). In the 20 ms mean jitter condition, the signal on each trial was shifted later by a latency selected randomly from a rectangular distribution between 0 ms and 40 ms. Likewise, in the 40 ms mean jitter condition the ERP was shifted later in time by a latency selected from a distribution ranging from 0 ms to 80 ms. Importantly, in the Distraction Decoherence model the gain of the signal remained constant (i.e. 100%) for every trial.

A data set was simulated, which consisted of 10 sets of 100 trials each. The simulated ERP was visualized by averaging across these 1000 trials at each level of modulation. Phase distribution, inter-trial phase coherence, total power, evoked power and induced power were computed as in Experiment Two.

### Results

**Figure 4:**
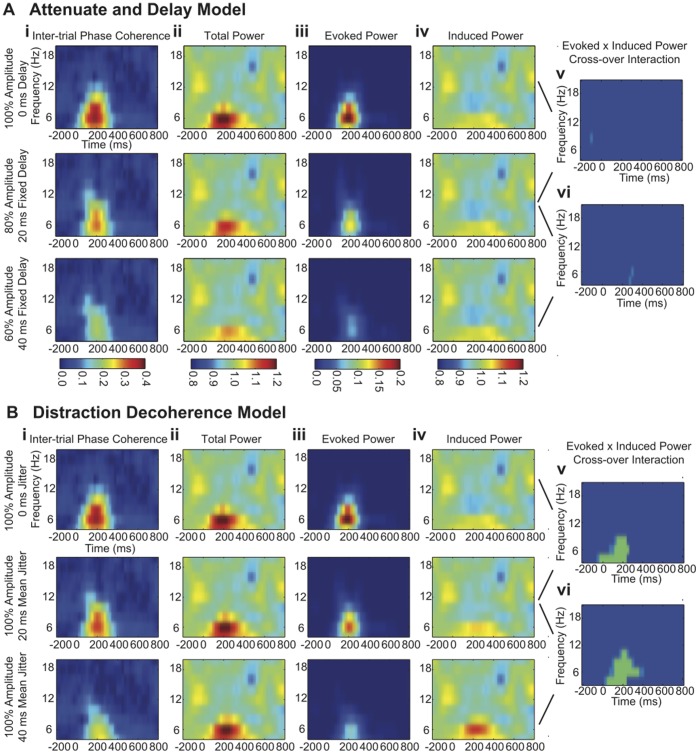
Time-frequency analysis of the Attenuate and Delay and Distraction Decoherence models. **4A)** Attenuate and Delay Model: time-frequency plots of (**i**) inter-trial phase coherence (**ii**) total power (**iii**) evoked power and (**iv**) induced power for the 100% amplitude/0 ms delay, 80% amplitude/20 ms fixed delay, and 60% amplitude/40 ms fixed delay modulations, respectively. (**v, vi**) Wilcoxen Rank Sum test masked for time-frequency bins that showed a significant directional cross-over interaction between evoked power and induced power: (**v**) compares 100% amplitude/0 ms delay to 80% amplitude/20 ms fixed delay and (**vi**) compares 80% amplitude/20 ms fixed delay to 60% amplitude/40 ms fixed delay. Light blue indicates time/frequency bins with p-values between 0.05 and 0.01 and green indicates bins with p-values less than 0.01. **4B)** Distraction Decoherence Model: time-frequency plots of (**i**) inter-trial phase coherence (**ii**) total power (**iii**) evoked power and (**iv**) induced power for the 100% amplitude/0 ms delay, 100% amplitude/20 ms mean jitter and 100% amplitude/40 ms mean jitter modulations. (**v, vi**) Wilcoxen Rank Sum test masked for time-frequency bins that showed a significant directional cross-over interaction between evoked power and induced power: (**v**) compares 100% amplitude/0 ms delay to 100% amplitude/20 ms mean jitter and (**vi**) compares 100% amplitude/20 ms mean jitter to 100% amplitude/40 ms mean jitter. Note that the test for the cross-over interaction between evoked power and induced power selectively identifies the phase jitter built into the Distraction Decoherence model without falsely finding phase jitter in the Attenuate-and-Delay model.

Figures 3A and 3B depict the ERP waveforms (absent noise for clarity) for each level of modulation under both models. In both models, the N1 peak is attenuated and shifted later in time. The latency shift is also evident when the phase distribution at the N1 latency is plotted in a radial phase histogram (Fig. 3C and Fig. 3D). Note that under both models, the distribution of phases is broadened and the phase distribution is rotated counter-clockwise. Likewise both models exhibit progressively reduced inter-trial phase coherence (Fig. 4A(i); Fig. 4B(i)). In the Attenuate-and-Delay model the distribution of phases is broadened because the ERP signal becomes progressively weaker relative to the noise background, which has random phase. In the Distraction Decoherence model the distribution of phases is broadened because the ERP signal itself is jittered in time.

We found that unlike inter-trial phase coherence, total power does differentiate the two models: as expected, the Attenuate-and-Delay model reduces total power, whereas the Distraction Decoherence model does not (Fig. 4A(ii); Fig. 4B(ii)). This difference in total power can be further explored by separately considering evoked and induced power [43]. In the Attenuate-and-Delay model, the ERP signal is always perfectly time-locked to time zero, albeit with increasingly longer latencies. Thus, the modulation in total power is due entirely to a reduction in evoked power (Fig. 4A(iii); Fig. 4A(iv)). By contrast, in the Distraction Decoherence model, the ERP signal becomes progressively less time-locked to time zero with increasing levels of jitter. Thus, although the total power in the signal remains constant, it shifts from evoked to induced power (Fig. 4B(iii); Fig. 4B(iv)).

Our simulation effectively replicates aspects of David et al. [44] and suggests a novel approach to detecting the signature of signal jitter in the ERP. Signal jitter is uniquely indicated by a directional cross-over interaction between evoked and induced power. We use the term '*directional cross-over interaction'* below to describe the specific characteristic changes in power that occur when a signal is jittered across successive trials. It is 'directional' in the sense that increasing jitter causes evoked and induced power to change in specific directions. It is a 'cross-over interaction' in that these quantities vary inversely. For example, increasing jitter causes evoked power to decrease while causing induced power to increase. Thus a directional statistical test for time/frequency bins that exhibit both a significant reduction in evoked power and a significant increase in induced power should reveal the presence of signal jitter without being confounded with amplitude modulation. To this end we applied a Wilcoxon signed-rank test across the median power values in our ten 100-trial data sets. In this way we independently compared both evoked power and induced power at different levels of modulation. For visualization, we masked time/frequency bins that did not fulfill the following criteria: 1) both induced and evoked power changed significantly according to the Wilcoxon test and 2) induced and evoked power change oppositely and in the predicted direction (i.e. increasing jitter reduces evoked power and increases induced power). Thus Figure 4 identifies bins that fit the characteristic power differences when two levels of inter-trial phase coherence are compared (Fig. 4A(v); Fig. 4A(vi); Fig. 4B(v); Fig. 4B(vi)). In addition, directional cross-over interactions successfully identified theta/alpha-band signal jitter in the Distraction Decoherence model, without spuriously suggesting jitter in the Attenuate-and-Delay model (Fig. 4A(v); Fig. 4A(vi); Fig. 4B(v); Fig. 4B(vi)).

**Figure 5:**
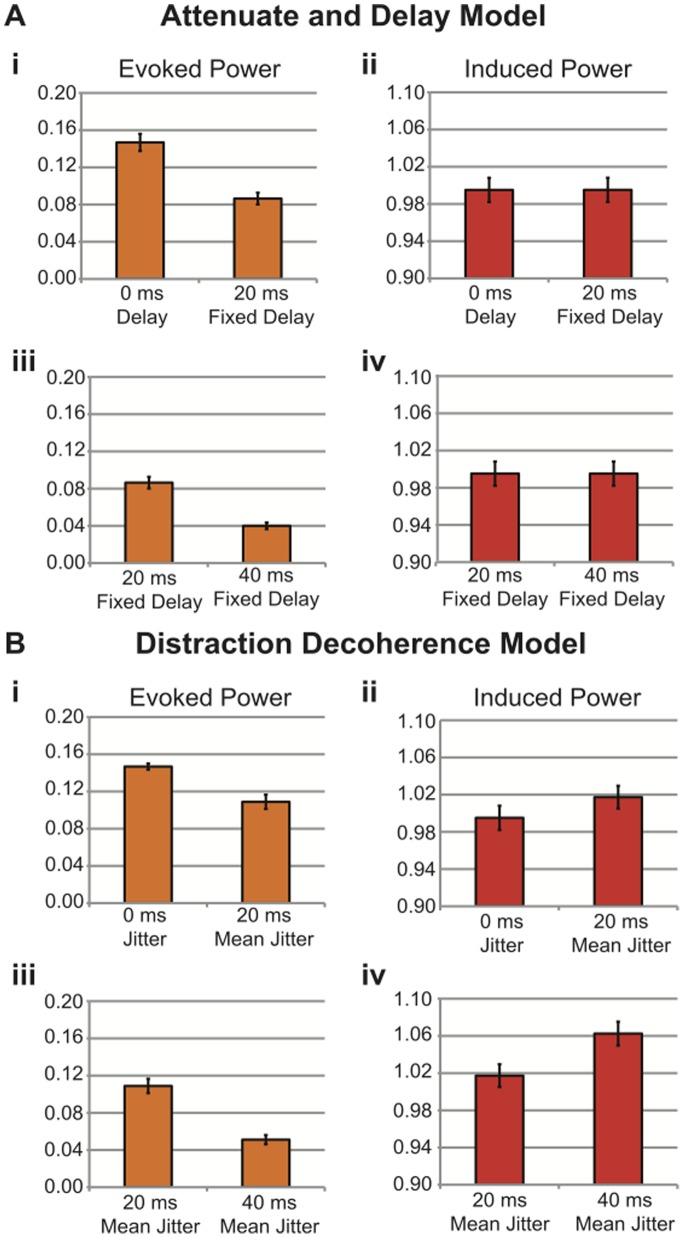
Directional Cross-Over Interactions Differentiate Attenuate and Delay from Distraction Decoherence Models. **5A) Attenuate and Delay Model:** Grand-averaged evoked (**i**) and induced (**ii**) power (125 ms to 225 ms and from 4 Hz to 8 Hz) at 100% amplitude/0 ms delay and 80% amplitude/20 ms fixed delay modulations. Grand-averaged evoked (**iii**) and induced (**iv**) power (125 ms to 225 ms and from 4 Hz to 8 Hz) at 80% amplitude/20 ms fixed delay and 60% amplitude/40 ms fixed delay modulations; error bars indicate the standard error of the mean. **5B) Distraction Decoherence Model:** Grand averaged evoked (**i**) and induced (**ii**) power (125 ms to 225 ms and from 4 Hz to 8 Hz) at 100% amplitude/0 ms delay and 100% amplitude/20 ms mean jitter modulations. Grand averaged evoked (**iii**) and induced (**iv**) power (125 ms to 225 ms and from 4 Hz to 8 Hz) at 100% amplitude/20 ms mean jitter and 100% amplitude/40 ms mean jitter modulations. Note the presence of an evoked power by induced power directional cross-over interaction in the Distraction Decoherence Model but not in the Attenuate and Delay Model.

The relationship between evoked and induced power in the directional cross-over interaction is further made evident in Figure 5. Mean values of evoked and induced power for fifteen time-frequency bins that passed our criteria for the directional cross-over interaction in the Distraction Decoherence model (125 ms to 225 ms and from 4 Hz to 8 Hz) were averaged to create a grand average of evoked and induced power at all three levels of modulation in the Distraction Decoherence model and in the Attenuate and Delay model for reference. The directional cross-over interactions between evoked and induced power successfully identified theta/alpha-band signal jitter in the Distraction Decoherence model and not in the Attenuate and Delay model (Fig. 5A(i); Fig. 5A(ii); Fig. 5A(iii); Fig. 5A(iv); Fig. 5B(i); Fig. 5B(ii); Fig. 5B(iii); Fig. 5B(iv)) (Note the absence of an increase in induced power in the Attenuate and Delay model). Therefore, we concluded that this directional cross-over interaction test is a suitable test to apply to our empirical data.

**Figure 6:**
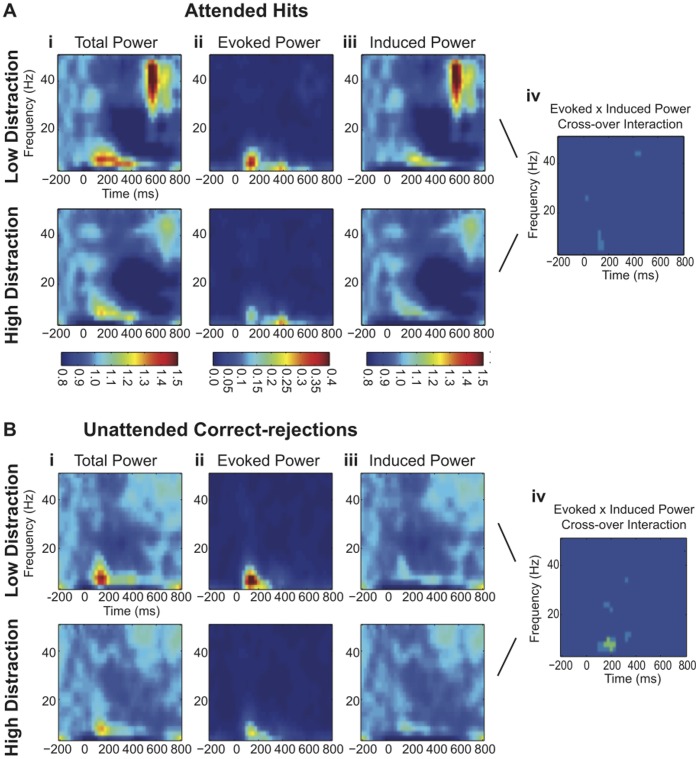
Decoherence Due to Distraction. **6A)** Time frequency plots of (**i**) total power (**ii**) evoked power and (**iii**) induced power for Attended Hits in low (above) and high (below) distraction. (**iv**) Wilcoxen Rank Sum maps masked to show bins exhibiting a significant directional cross-over interaction between evoked and induced Power. Light blue indicates time/frequency bins with p-values between 0.05 and 0.01 and green indicates bins with p-values less than 0.01. **6B)** Time frequency plots of (**i**) total power (**ii**) evoked power and (**iii**) induced power for Unattended Correct-rejections in low (above) and high (below) distraction. (**iv**) Wilcoxen Rank Sum maps masked to show bins exhibiting a significant directional cross-over interaction between evoked power and induced power. Note the significant crossover interaction in the theta/alpha band at the N1 latency range, particularly for Unattended Correct-rejections.

**Figure 7:**
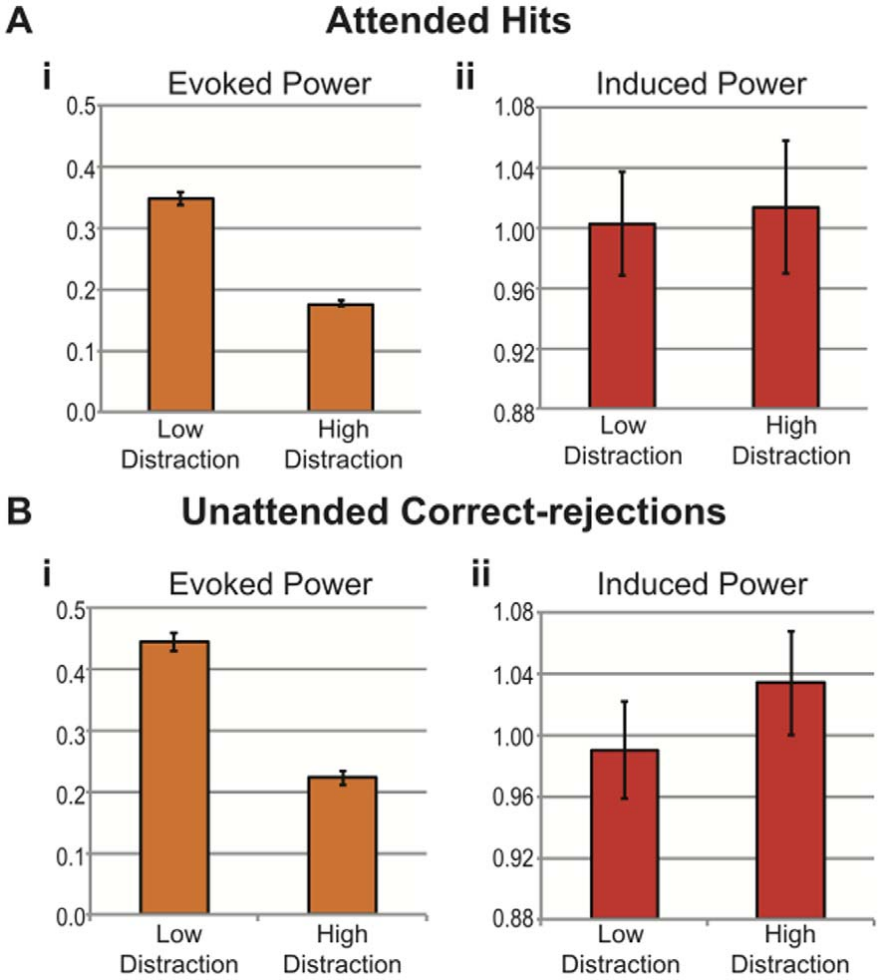
Evoked Power by Induced Power Directional Cross-over Interaction due to Distraction. **7A)** Grand-averaged evoked (**i**) and induced (**ii**) power in low- and high-distraction for Attended Hits at time-frequency bins: 125 to 150 ms; 6 to 8 Hz; error bars indicate the standard error of the mean. **7B)** Grand-averaged evoked (**i**) and induced (**ii**) power in low- and high-distraction for Unattended Correct-rejections at time-frequency bins: 125 to 150 ms; 6 to 8 Hz. Note that both Attended Hits and Unattended Correct-rejections show evidence of a directional evoked power by induced power cross-over interaction.

Figure 6 shows the results of applying the directional cross-over interaction test to the data collected in Experiment Two for Attended Hits and Unattended Correct-rejections. Note that in both cases the total power and evoked power is reduced under high relative to low-distraction (Fig. 6A(i); Fig. 6B(i); Fig. 6A(ii); Fig. 6B(ii)). Also, substantial alpha suppression is evident for the Attended Hit condition (Fig. 6A(i); Fig. 6B(i)) but this did not reach significance as revealed by FDR correction. Importantly, a directional crossover interaction is evident in the theta/alpha band at a latency range spanning the N1 and P2 components, particularly for Unattended Correct-rejections at the non-target (ignored) frequency (Fig. 6A(iv); Fig. 6B(iv)), thereby indicating the presence of signal jitter in the ERP. Mean values of evoked and induced power for 4 time-frequency bins (125 to 150 ms and from 6 to 8 Hz) that passed criteria for the directional cross-over interaction for both Attended Hits and Unattended Correct-rejections were averaged to create a grand average of evoked and induced power in both low- and high-distraction for each condition (Fig. 7A(i); Fig. 7A(ii); Fig. 7B(i); Fig. 7B(ii)). For both Attended Hits and Unattended Correct-rejections evoked power decreased and induced power increased in the high relative to low-distraction conditions, respectively. Note that baseline correction was performed for visualizing power changes in Figure 6 as percent change from baseline, but the cross-over interaction is computed without baseline correction to avoid potentially confounding effects of temporal blurring of power from post- to pre-stimulus bins.

### Discussion

Experiment Three demonstrated that attenuation of an ERP component could result from a simple jittering of that component across trials. *This applies, in principle, to all studies that make use of the ERP technique.* Attenuation of amplitude in the ERP does not unequivocally indicate gain modulation. By contrast, a reduction of total power does seem to indicate gain modulation. Likewise, inter-trial phase coherence is a sensitive but not specific indicator of signal jitter. Modulation of inter-trial phase coherence does not unequivocally indicate modulation of phase. By contrast, an evoked x induced directional cross-over interaction does seem specific to signal jitter. Importantly, these measures are not mutually exclusive. For example, we proposed two models to account for our data: Attenuate-and-Delay and Distraction Decoherence. Our data exhibit both a reduction of total power and an evoked x induced directional cross-over interaction. We conclude therefore that high-distraction both attenuates gain and jitters the evoked signal on individual trials. In particular, the effect of distraction on phase variability appears to be stronger for tones occurring at an unattended frequency (Fig. 6A(iv); Fig. 6B(iv)) suggesting that focused attention may prevent Distraction Decoherence. In addition, there appears to be substantially more alpha suppression in the Attended Hit condition as compared to the Unattended Correct-rejection condition (Fig. 6A(i); Fig. 6B(i)). It is likely that this alpha suppression is reflective of increased attentional demands due to the fact that the “attended” tones were also task-relevant targets that required a response [45], [46].

## General Discussion

In this study we investigated the physiological correlates of auditory distraction. We found that, relative to broad-band noise, the presence of a continuous speech distractor significantly impaired pitch discrimination of a pair of target tones. We also replicated early attenuation of ERP components previously observed in various studies [14], [15], [16], [17], [18], [27] as well as the reduction in inter-trial phase coherence reported by Ponjavic-Conte et al. [27]. As predicted, high-distraction attenuated and delayed the N1 peak evoked by target and non-target stimuli. High-distraction also had a strong effect on theta/alpha band inter-trial phase coherence around the N1 latency for both Attended Hits and Unattended Correct-rejections.

Experiment Three considered two explanations for the N1 attenuation, N1 delay and associated reduction of inter-trial phase coherence. We found that two models could account for the data: one in which distraction attenuates and delays a fixed-latency ERP component (Attenuate-and-Delay Model) and one in which the latency of an ERP signal is “jittered” in time across trials (Distraction Decoherence Model). However, we found that the existence of signal jitter across trials is revealed unequivocally by a directional cross-over interaction between evoked and induced power. The data in experiment two exhibited precisely this directional cross-over interaction, suggesting that signal jitter is an important consideration in understanding the effects of distraction. However, distraction also reduced total power, suggesting that gain attenuation [28] is also a correlate of distraction.

In addition to auditory masking interpretations (see Experiment Two) the present results can also be interpreted in the context of selective attention. The gain-control theory of attention holds that attention acts to modulate the gain of fixed-latency responses in sensory systems [28], [29], [30]. The earliest effects of auditory attention (the early negative difference or “early ND”) require that attention be *sustained* at a given frequency or location for several tens of seconds [21], [22]. The early ND is maximal at fronto-central sites and is believed to reflect modulation of auditory cortex on the supratemporal plane [30]. When attention is re-oriented on a moment-by-moment basis, as in cue-target [23], [25] or target-target [24] paradigms, the earliest effect of attention occurs after the N1 peak; thus later than in the sustained attention case (but see [26] for contrasting data). This modulation has been called the Nd1 and is maximal over posterior-contralateral scalp sites suggesting modulation in a posterior “where” auditory pathway [25]. The differences between the effects of sustained and transient attention on the ERP suggest that top-down attentional set takes time to deploy, at least at early stages of auditory processing.

If distraction transiently and repeatedly captures one’s attention away from a stream of target tones, then attention would be operating in a transient rather than sustained mode, and the boost of early ERP components due to attention would be prevented. In this sense, distraction is conceptually the opposite of attention. This is possibly why “low” compared to “high” distraction ERP waveforms in the present study qualitatively resemble “attended” and “unattended” stimuli in previous attention studies [19]. Note however that there is a fundamental difference between the distraction paradigm employed here and the sustained-attention paradigm used by Hillyard and colleagues [19]. In the present study, the target and non-target tones never changed in pitch or location throughout the session. Only the kind of distractor was changed across blocks of trials. That is, the top-down goal of the listener was to maintain a constant attentional set with respect to the target stimuli. The differences in ERP waveforms can be seen as reflecting an involuntary breakdown of attentional set under high compared to low-distraction. However, our data show no evidence of a reorienting negativity (RON), [47] which might be expected if attention is being shifted and re-shifted during distraction. It is possible that some activity related to reorienting may not have been clearly visible because of signal jitter due to distraction. Furthermore, because our distractor stimuli consisted of continuous speech rather than discrete stimuli or unusual events typically used to study auditory distraction [7], [48], we were unable to extract ERP waveforms associated with distractors. However, we point out that our choice of stimuli were ecologically valid and were used in an attempt to capture the general effect of real-world distraction in situations such as the two-talker problem.

The simulations in Experiment Three demonstrate a principle of substantial general importance. Differential signal jitter across trials can account for what appears to be amplitude modulation of components in the averaged ERP. This is a familiar idea in the ERP field: jitter between temporally adjacent stimuli is often introduced *by design* to reduce the overlap of ERP waveforms. For example, in cue-target paradigms it is common practice that the cue-target onset asynchrony is randomly distributed across a range of several hundred milliseconds. Here we extend the concept to apply to signals within an epoch of interest. When the activity of a neural circuit becomes decoupled in time from the sensory events that trigger it, its signal becomes attenuated in the ERP. This can occur even when the true amplitude of that signal does not change from trial to trial. This observation can explain why, for example, Hari and Makela [16] found that speech or music maskers attenuate the N1m, but that this amplitude reduction was not associated with substantial impairment of perception. Our model of Distraction Decoherence shows that the N1/N1m in this case may indeed have been triggered on each trial, but simply jittered in time. In another example, Dowdall et al. [49] showed that the well-known N2pc component of the visual evoked potential is present on pop-out search trials but appears absent on non-popout search trials. They found that the N2pc on non-popout trials is simply jittered relative to the onset of the search array, whereas the N2pc on popout trials exhibits good inter-trial phase coherence and is therefore visible in the ERP.

We next consider some possible mechanisms of Distraction Decoherence. One possibility is that Distraction Decoherence arises because a subset of neural ensembles becomes phase locked to amplitude modulation of the speech signal in the high-distraction condition. Speech has an envelope of amplitude modulation that fluctuates approximately at the theta frequency, and this envelope is known to be tracked in the auditory EEG signal [50]. A simple explanation might be that this extra activity injects phase noise into the ERP. However this is unlikely because the baselines did not differ in induced power across conditions as would be expected if additional signal was present throughout high-distraction blocks.

Another view of Distraction Decoherence considers that it may not be possible for the auditory system to both track the phase of a competing speech signal and respond consistently to occasional events such as our target tones. One view of the ERP signal is that it reflects transient phase reorganization and consolidation of ongoing oscillations in the EEG [51], [52], [53], [54], [55], [56] although some reported data are also found to be more consistent with an additive fixed-latency view of ERP generation [57]. It may be that distraction disrupts the timing of such phase resetting that would normally exhibit high inter-trial coherence. Inter-trial phase coherence might reflect a mechanism that attempts to entrain to a periodic environmental stimulus as a means of attentional selection [37], [38]. For example, Schroeder & Lakatos [58] proposed that the brain might act in two modes with respect to attention: a “vigilance” mode characterized by readiness to respond to discrete events in time, and a “rhythmic” mode characterized by phase entrainment with a to-be-attended periodic signal. The brain cannot effectively be in both modes at once. Distraction Decoherence might occur because the high-distraction speech signal causes the brain to enter a rhythmic mode. To respond to the temporally unpredictable occurrence of the probe tones, the brain would need to escape this rhythmic mode and switch to the vigilance mode. Since the distracting speech, and therefore any entrained oscillation in the auditory system, could have any phase at the moment of target onset, this switching might take slightly different amounts of time on different trials, thereby jittering the subsequent ERP response.

### Conclusion

Distraction is a common occurrence in any complex sensory environment. Although much is known about related attentional processes and their physiological correlates, little is known about the consequences of distraction itself. The present study showed that distraction leads to attenuation of the gain with which the auditory system responds to probe tones. We also showed that distraction disrupts the time-locking of neural responses relative to acoustic events in the environment. We propose the term *Distraction Decoherence* to describe the resulting breakdown in coherence of the EEG signal across successive trials. In general, the concept of inter-trial phase decoherence could account for a wide variety of situations in which a cognitive or perceptual manipulation leads to an apparent attenuation of a component in the averaged ERP waveform. The exact reasons why Distraction Decoherence occurs, and the mechanistic significance of inter-trial phase coherence in general, remain to be explored.
