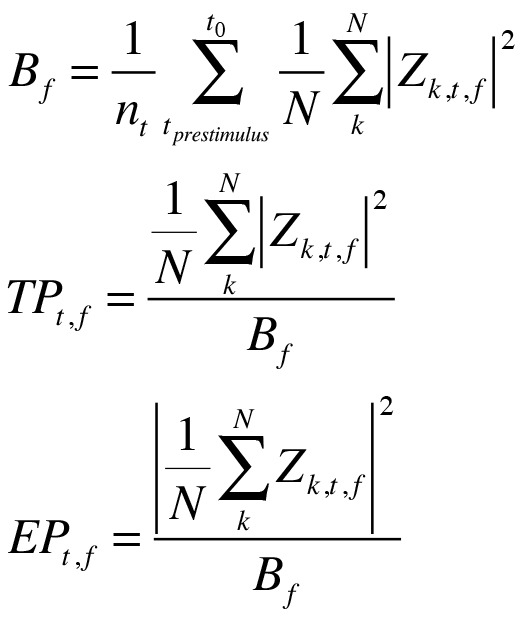# Correction: Dynamics of Distraction: Competition among Auditory Streams Modulates Gain and Disrupts Inter-Trial Phase Coherence in the Human Electroencephalogram

**DOI:** 10.1371/annotation/631b811c-5665-4b9d-9b3a-d5e41284f2b2

**Published:** 2013-10-23

**Authors:** Karla D. Ponjavic-Conte, Dillon A. Hambrook, Sebastian Pavlovic, Matthew S. Tata

In the Methods section of Experiment Two, there is an error in the third, fourth and fifth equations. The coefficient of the complex valued result (A_k,t,f_) should be replaced by the complex valued result (Z_k,t,f_). Please view the complete, correct, equations here: